# How to Administer Quinine in Malarial Intermittent Fevers

**Published:** 1886-11-20

**Authors:** 


**How to Administer Quinine in Malarial Intermittent Fevers.**—The following, though not new, has proved of such good service in several hundred cases of malarial fevers which came under our care, that we deem it of sufficient importance for publication. This way of administering the anti-periodic was suggested to us a number years ago by our friend Dr. H. D. Schmidt, who claims to have gotten it from the late Prof. G. B. Wood, of Philadelphia : In intermittent fever always give the quinine in the intermission, the first dose twelve hours before the expected paroxysm, a second dose six hours after the first, and a third dose three hours after the second. Given in this way, the dose needs seldom exceed 5 grains.—*N. O. Med. & Surg. Journal.*

